# Recombinant Marek’s disease virus type 1 provides full protection against very virulent Marek’s and infectious bursal disease viruses in chickens

**DOI:** 10.1038/srep39263

**Published:** 2016-12-16

**Authors:** Kai Li, Yongzhen Liu, Changjun Liu, Li Gao, Yanping Zhang, Hongyu Cui, Yulong Gao, Xiaole Qi, Li Zhong, Xiaomei Wang

**Affiliations:** 1 Avian Immunosuppressive Diseases Division, State Key Laboratory of Veterinary Biotechnology, Harbin Veterinary Research Institute, Chinese Academy of Agricultural Sciences, Harbin 150069, P.R. China

## Abstract

Marek’s disease virus (MDV) is a preferred vector in the construction of recombinant vaccines. However, bivalent vaccine based on MDV that confers full protection against both very virulent Marek’s and infectious bursal disease virus (IBDV) infections in chickens has not been produced. Here we developed a system utilizing overlapping fosmid DNAs transfection that rescues an MDV type 1 (MDV1) vaccine strain. Using this system, we inserted the IBDV VP2 gene at MDV1 genome sites UL41, US10 and US2. The VP2 protein was stably expressed in the recombinant MDV-infected cells and self-assembled into IBDV subviral particles. Insertion of the VP2 gene did not affect the replication phenotype of MDV in cell cultures, nor did it increase the virulence of the MDV vaccine strain in chickens. After challenge with very virulent IBDV, r814US2VP2 conferred full protection, whereas r814UL41VP2 and r814US10VP2 provided partial or no protection. All the three recombinant vaccines provided full protection against very virulent MDV challenge in chickens. These results demonstrated that r814US2VP2 could be used as a promising bivalent vaccine against both Marek’s and infectious bursal diseases in chickens.

Marek’s disease (MD) is a neoplastic and neuropathic disease in chickens that was first reported by Joszef Marek over a century ago^1^. Marek’s disease virus (MDV) strains have three serotypes: serotype 1 (MDV1) includes all the pathogenic strains and the attenuated strains of these viruses; serotype 2 (MDV2) includes naturally non-pathogenic strains; and serotype 3 (MDV3) is represented by turkey herpes virus (HVT)^2^. MDV1 remains the only neoplastic disease for which an effective vaccine has been used successfully and widely^3^. MDV has a large genome which consists of a unique long (UL) region and a unique short (US) region, both flanked with repeat sequences. The MDV genome has several regions that are nonessential for viral replication and therefore, suitable for the insertion of foreign genes, rendering the MDV1 vaccine strains a desirable live virus vector for expressing foreign genes^4^,^5^,^6^,^7^.

Infectious bursal disease (IBD) is an acute contagious immunosuppressive disease of young chickens caused by infectious bursal disease virus (IBDV)^8^. Since the discovery of the classic strains during the first outbreak of IBD in 1957, antigenic variants and very virulent IBDV (vvIBDV) strains have emerged^9^,^10^, which represented new challenges for effective prevention of IBD. Since IBDV causes disease in young chickens, early immunization is important for the prevention of the viral infection. However, with the high levels of circulating maternal antibodies, the immunity of attenuated live vaccines of IBDV can be easily inhibited^11^. To overcome the maternal antibodies, medium virulent vaccines of IBDV were used, which induced better protection than the attenuated vaccines, however, at a cost of inducing bursal damage and the failure of immunity of other poultry vaccines^12^. In addition, the virulence of live vaccines could be increased after passages in chickens^13^. Therefore, it is necessary to develop safer and more efficacious vaccines to prevent vvIBDV infection. The strictly ordered part of the IBDV capsid is made exclusively by VP2^14^, which is the major protective antigen of IBDV and contains the epitopes that are responsible for eliciting neutralizing antibodies^15^. Considering its lower susceptibility to maternal antibodies and good safety, recombinant MDV1 vaccines expressing VP2 would be more desirable than the conventional IBDV live vaccines.

In the early 1970s, HVT vaccine was firstly introduced in the field for the control of MD^3^. After a decade of use, very virulent MDV (vvMDV) strains emerged and began to break through the HVT protection, prompting the introduction of a more effective bivalent vaccine that consisted of the MDV2 SB-1 strain plus HVT^16^. However, by the early 1990s, very virulent plus MDV (vv+MDV) strains began to emerge and overcome protection provided by bivalent vaccines^16^. Currently, MD control was achieved by using the attenuated MDV1 vaccines such as CVI988 which are proving to be probably the only effective vaccine against some of currently prevalent vv and vv+MDV strains. The attenuated MDV1 strain, 814, was introduced in China since 1980s; this strain has been widely used in China as an important live vaccine for the prevention of high virulent MDV infection with a proven record of efficacy and safety^17^,^18^,^19^. Here, we demonstrate a system for generating the 814 strain by transfecting overlapping fosmid DNAs. Using this system, the IBDV VP2 gene was inserted at different sites in MDV1 genome. The recombinant virus r814US2VP2 containing VP2 gene at US2 site confers full protection against vvMDV and vvIBDV infection in chickens. These results advance the development of efficient recombinant MDV vaccines and rapid manipulation of the viral genome for basic research.

## Results

### Construction of an overlapping fosmid system for MDV reconstitution

The intact MDV genomic DNA (~170 kb) was purified from cells infected with strain 814 (Fig. S1A). After the genomic DNA was sheared and end-repaired, 36~48 kb fragments were purified from the agarose gels. These fragments were inserted into fosmid vector pCC1FOS, generating an MDV fosmid library. The size of each DNA fragment was approximately 40 kb, based on results of a NotI digestion of the recombinant fosmids (Fig. S1B). After end sequencing, 24 fosmids with MDV genomic DNA insertions were selected for virus rescue; the size and location of these DNA fragments in the 814 genome are shown in Table S1. For virus rescue, 10 sets of fosmid clones, each of which consisted of five- or six-fosmid combinations covering the entire MDV genome, were transfected into primary CEFs (Table S1). After five days of transfection, all of the 10 sets of fosmid combination-transfected cells exhibited MDV1 typical cytopathic effects (CPE). The transfection of set 2 resulted in the highest percent of plaques, therefore, this combination was used in further studies; virus rescued from this set was designated rMDV.

### Characterization of the rescued parental MDV vaccine virus

**Figure 1:**
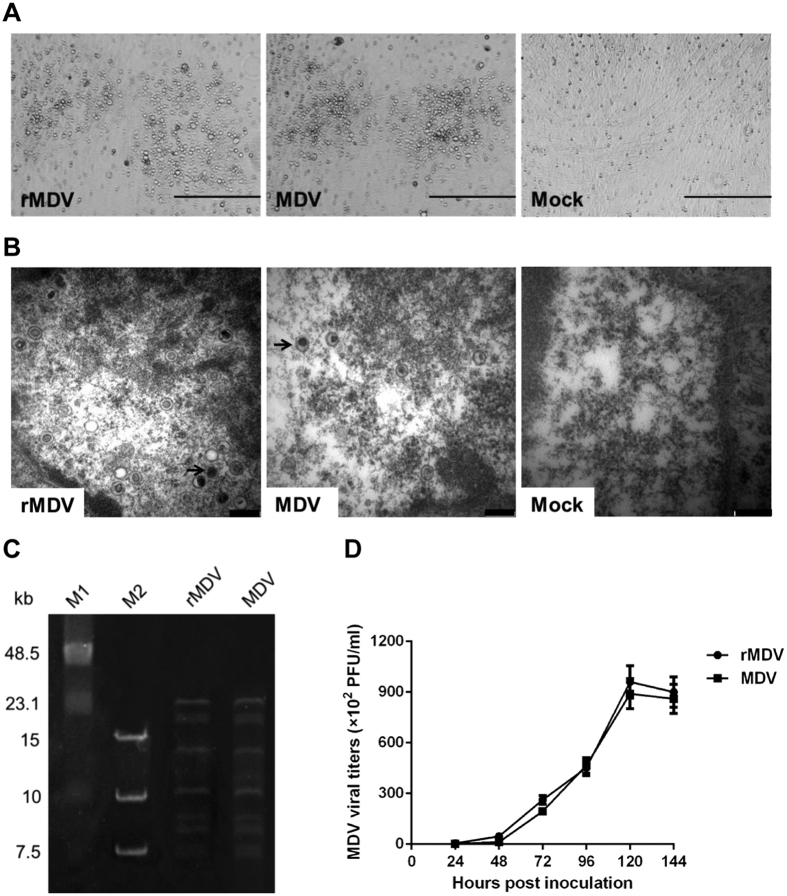
Characterization of the rescued parental MDV vaccine virus (rMDV) and the wild-type virus (MDV). (**A**) The cytopathic effects induced by rMDV and MDV. Bar length, 400 μm. (**B**) Electron microscopy detection of rMDV and MDV in infected CEFs. Bar length, 200 nm. Arrows represent the MDV virions detected in the cell nucleus in infected cells. (**C**) The restriction pattern of the rMDV and MDV genomic DNA digested with HindIII. (**D**) The growth properties of rMDV and MDV in cell cultures. Data presented are the means ± standard deviations (S.D.) from three independent experiments.

The rescued parental vaccine virus rMDV was characterized and compared with the wild-type virus. The rescued virus yielded plaques similar to those resulting from the wild-type virus (Fig. 1A), and generated in the nucleus multiple naked virions indistinguishable in size and shape from those of wild-type virus (Fig. 1B). Pulsed field gel electrophoresis analysis of the rescued and wild-type vaccine virus DNA digested with HindIII showed similar restriction patterns, indicating no rearrangement in the rescued viral DNA (Fig. 1C). *In vitro* characterization of rMDV also showed that the growth properties of this rescued virus were similar to those of the wild-type vaccine virus (Fig. 1D). These results indicate that we successfully rescued the MDV1 vaccine strain 814 using overlapping fosmid DNAs, and that the rescued viruses had a genotype and replication phenotype similar to those of wild-type virus.

### Generation of recombinant MDV containing IBDV VP2 gene

**Figure 2:**
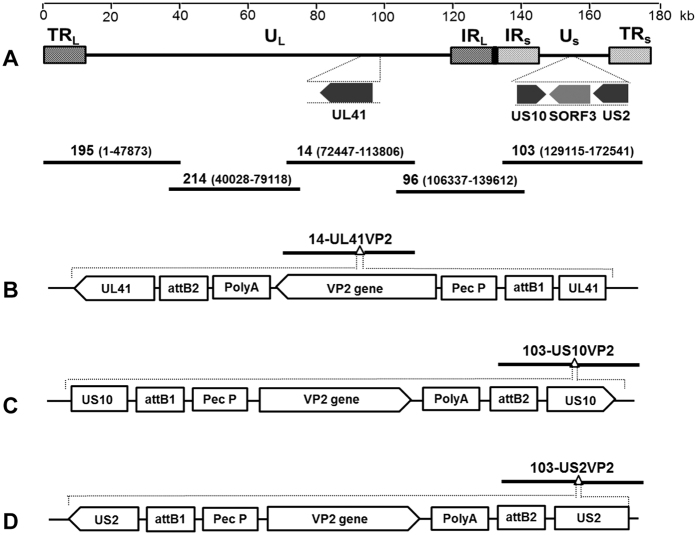
Construction of recombinant fosmids inserted the VP2 gene of IBDV. **(A)** The genomic structure of MDV, and the five fosmids derived from MDV1 vaccine strain 814 used for MDV regeneration. Numbers represent the fosmid names and the location of each fosmid fragment in the genome of strain 814. **(B–D)** The schematic diagrams of recombinant fosmids inserted the VP2 gene of IBDV within the UL41 (**B**), US2 (**C**) and US10 (**D**) genes.

We next inserted the VP2 gene of vvIBDV strain HLJ0504 into the UL41, US10 and US2 sites in the MDV1 vaccine genome for the construction of recombinant vaccines against IBDV. We firstly constructed a VP2-expressing cassette by cloning the VP2 gene under the control of the Pec promoter, and then subcloned the cassette into an entry vector with the attL1 and attL2 sequences. Finally, the VP2 expressing cassette was inserted into the above sites in the modified fosmids by LR reaction. The resultant fosmids containing *VP2*, 14-UL41VP2, 103-US10VP2, and 103-US2VP2 (Fig. 2B–D), were co-transfected with the other four parental fosmids for virus rescue. The MDV1-typical plaques observed in the CEFs and the virions detected in the electron microscopy analysis indicated that recombinant MDVs expressing *VP2* were rescued successfully (Fig. S2).

### Biological characterization of the recombinant MDV expressing VP2

**Figure 3:**
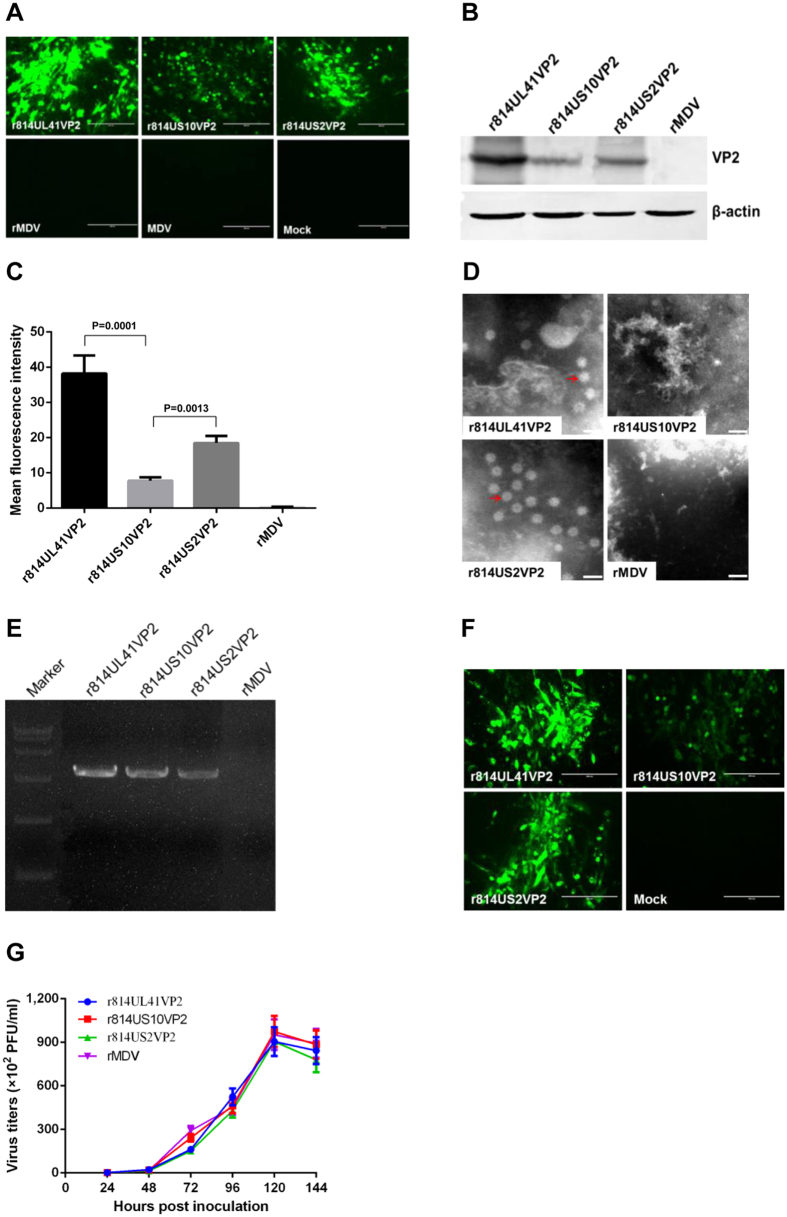
Characterization of the recombinant MDVs expressing VP2 gene. **(A)** Confirmation of the successful expression of VP2 by indirect immunofluorescence assay. **(B)** Detection of the expression of VP2 with western blotting. Chicken β-actin was detected as the internal control. **(C)** Comparison of VP2 expression from different viruses with FACS analysis. Results presented as the mean fluorescence intensity (MFI) of the VP2 positive cells in 10,000 events captured during the procedure. **(D)** Negative-stain electron microscopy detection of the IBDV subviral particles expressed by rMDV-VP2. Bar length, 40 nm. **(E)** PCR amplification of the VP2 gene from the recombinant viruses passaged 20 times in CEFs. **(F)** Detection of VP2 expression from the recombinant viruses passaged 20 times in CEFs with indirect immunofluorescence assay. **(G)** Comparison of the replication kinetics of the recombinant viruses. Data presented are the means ± standard deviations (S.D.) from three independent experiments. Statistical significance was set at *P* < 0.05.

*VP2* expression by the recombinant MDVs was confirmed by the presence of green fluorescent signal in the infected cells, as detected by immunofluorescence (Fig. 3A). Western blot analysis of rMDV-VP2-infected CEF lysate with anti-VP2 MAbs indicated that the molecular mass of the expressed VP2 was about 50 kDa (Fig. 3B). In both the immunofluorescence assay (IFA) and western blotting, r814UL41VP2 exhibited the strongest signal, followed by r814US2VP2; r814US10VP2 showed the weakest signal. VP2 expression was further quantified by fluorescence-activated cell-sorting (FACS); the results revealed that r814UL41VP2 produced the highest fluorescent value and r814US10VP2 showed the lowest value, while r814US2VP2 produced VP2 at a medium level (Fig. 3C). Negative-stain electron microscopy detected approximately 23 nm subviral IBDV particles in cells infected with r814UL41VP2 or r814US2VP2, but not in cells infected with r814US10VP2 or parental MDV (Fig. 3D).

PCR amplification and sequencing of *VP2* inserted in the recombinant MDVs passaged 20 times in CEFs confirmed that the recombinant MDVs had good genetic stability (Fig. 3E). VP2 expression from the serially passaged viruses was also confirmed by IFA with anti-VP2 MAbs (Fig. 3F). Further analysis demonstrated that the growth kinetics and magnitude of the recombinant MDVs expressing *VP2* were very similar to those of the parental virus (Fig. 3G), indicating that *VP2* insertion in the MDV genome did not affect the replication of the MDV vaccine strain.

### Antibody responses against IBDV induced by recombinant MDVs in chickens

**Figure 4:**
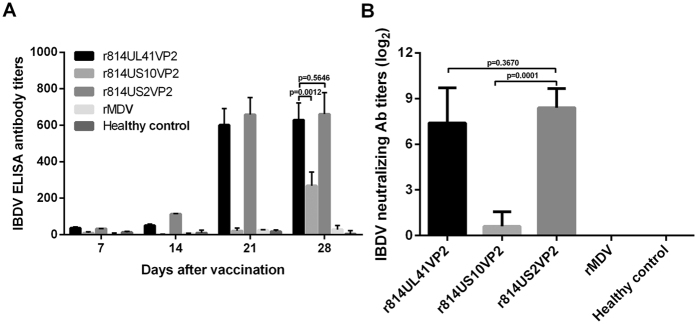
Antibody responses against IBDV in chickens. **(A)** Detection of IBDV antibody titres of immunized animals by ELISA. Sera were collected weekly and detected using a commercial IBDV Antibody test kit (IDEXX, Westbrook, Maine). **(B)** Detection of viral neutralization antibodies against IBDV in chickens after 28 days of vaccination. Data presented are the means ± standard deviations (S.D.) from 10 birds in each group. Statistical significance was set at *P* < 0.05.

We next tested the antibody responses against IBDV in specific-pathogen-free (SPF) chickens immunized with the recombinant vaccines. As shown in Fig. 4A, r814UL41VP2 and r814US2VP2 induced detectable enzyme-linked immunosorbent assay (ELISA) antibodies against IBDV from 3 weeks post vaccination (w.p.v), and the antibody titres induced by these two viruses before challenge at 4 w.p.v were comparable (*P* > 0.05). By comparison, r814US10VP2 induced detectable antibodies after 4 w.p.v, and the antibody levels were lower than those induced by r814UL41VP2 and r814US2VP2 (*P* < 0.05). Neutralizing antibodies against IBDV induced by the recombinant vaccines were detected at 4 w.p.v (Fig. 4B). The results showed that r814UL41VP2 and r814US2VP2 induced neutralising antibodies at comparable levels (336 and 378, respectively), which were significantly higher than that induced by r814US10VP2 (*P* < 0.05). As expected, chickens immunized with the parental virus rMDV did not induce any detectable antibodies against IBDV during the experiment.

### Protective efficacy against lethal vvIBDV challenge in chickens

**Figure 5:**
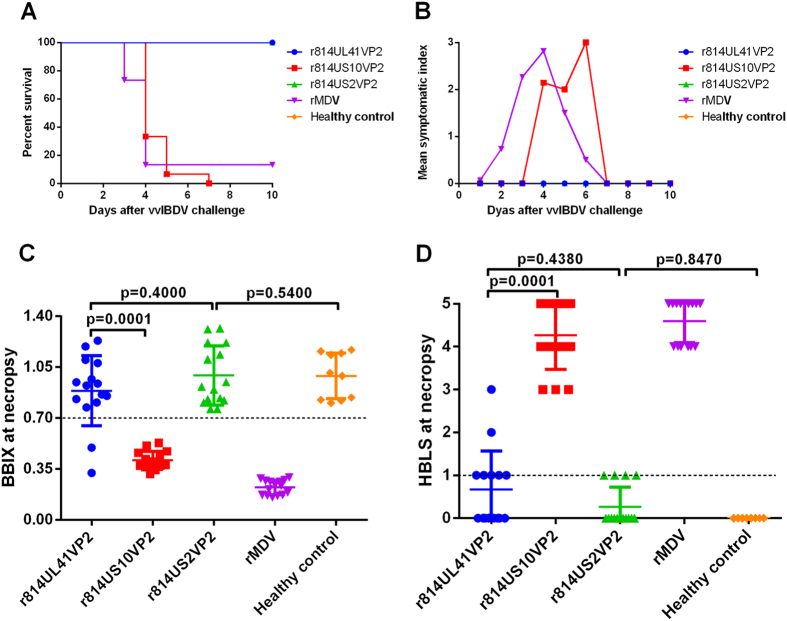
Protective efficacy of the recombinant vaccines against vvIBDV challenge. **(A)** Survival rate of chickens challenged with vvIBDV within an observation period of 10 days. **(B)** The mean symptomatic index scores for chickens after vvIBDV challenge. **(C)** The bursa: body-weight index (BBIX) values of chickens died and survived after challenge. **(D)** The histopathologic bursal lesion score (HBLS) of chickens died and survived after challenge. The dead birds were dissected at the day when they died and the surviving birds were sacrificed and analyzed after the observation period at day 10 post challenge. Data presented are the means ± standard deviations (S.D.) from 15 birds in each of the vaccinated group and 10 birds in the healthy control group. Statistical significance was set at *P* < 0.05.

To evaluate the protective efficacy of the recombinant viruses expressing VP2 against IBDV, vaccinated chickens were challenged with vvIBDV HLJ0504 at 4 w.p.v. All the chickens vaccinated with r814UL41VP2 and r814US2VP2 were free of clinical signs and mortality after the challenge (Fig. 5A and B). In contrast, all the chickens vaccinated with r814US10VP2 showed clinical signs of IBD, and died after challenge. Chickens immunized with the parental virus were fully susceptible to the IBDV challenge, showing either high mortality (87%) or severe clinical signs (100%), whereas the healthy control group did not exhibit these signs.

The bursa samples were collected after the observation period, and the bursa: body weight index (BBIX) and histopathological bursal lesion score (HBLS) were calculated in order to analyse the extent of bursal atrophy and histopathological lesions. As shown in Fig. 5C and D, all the chickens vaccinated with r814US2VP2 showed a BBIX value higher than 0.7 and a HBLS value no more than one, indicating that no bursa had atrophy or gross lesions in this group. Among the 15 surviving chickens in the r814UL41VP2 group, two chickens exhibited bursal atrophy with a BBIX value lower than 0.7, and these two chickens also showed mild bursal lesions (HBLS 2 and 3). The two surviving chickens in the rMDV group had severe bursal atrophy (BBIX 0.21 and 0.16) and gross lesions (HBLS 5), while no bursal atrophy and lesions were observed in the healthy controls (0/10). Together the results of mortality, clinical signs and bursal lesions, vaccination with r814UL41VP2, r814US10VP2, and r814US2VP2 conferred 87% (13/15), 0% (0/15), and 100% (15/15) protection against the lethal vvIBDV challenge.

### Protective efficacy against lethal vvMDV challenge in chickens

To determine if the insertion of *VP2* influenced the immunogenicity of the parental vaccine virus, chickens vaccinated with the recombinant MDVs as well as the parental vaccine virus were challenged with the vvMDV Md5 strain 7 days after inoculation and examined for another 12 weeks. After challenge, 85% (17/20) of the chickens in the unvaccinated challenge control group died of MD, and the remaining 3 chickens had histopathological MD lesions at the necropsy. The chickens vaccinated with the recombinant MDVs and the parental vaccine virus did not show clinical signs and had no gross or histopathological tumors, indicating that the recombinant MDVs conferred full protection to chickens against vvMDV infection.

## Discussion

The development of recombinant vaccines has been one of the most well studied aspects of molecular virology. MDV vaccine strain is a highly desirable live virus vector for expressing foreign antigen genes from other infectious viruses. In practice, only MDV vaccines can be inoculated into day-old field chicks that have high titres of maternal antibodies, because the virus is transmitted cell to cell and is less susceptible to maternal antibodies than some other virus vaccines^3^. Moreover, the MDV vaccines can induce lifetime immunity in chickens with just one vaccination^7^. In the previous studies, recombinant HVT and MDV1 vaccines expressing antigen genes from several poultry viruses such as avian influenza virus^7^,^20^, Newcastle disease virus^4^,^21^, infectious bronchitis virus^6^ and IBDV^5^,^22^, have been developed. However, with the emergence of vv and vv+MDV strains, HVT could no longer provide efficient protection against high virulent MDV infection^23^,^24^,^25^. By comparison, attenuated MDV1 vaccines could induce more effective protection against the prevalent vv and vv+MDV strains, probably due to closer antigenic similarity with the oncogenic strains^3^,^16^. From this point of view, attenuated MDV1 strains are more desirable than HVT as a viral vector for the development of bivalent recombinant vaccines against vvMDV infection and other poultry diseases.

In this study, three sites in the MDV1 vaccine genome were compared for the expression of *VP2*. The results revealed that the VP2 expression level from US2 was significantly higher than that from US10. These results are consistent with those of a previous study, which demonstrated that the *HA* gene from avian influenza virus exhibited a higher expression level from US2 than from US10 in recombinant MDV^7^. Although several nonessential genes for viral growth have been identified in MDV1 UL area, there are no reports of recombinant MDV1 containing foreign antigen genes in this area. In the present study, the VP2 gene was inserted into the UL41 gene without influencing the viral growth. The VP2 expression level from UL41 was shown to be significantly higher than those from the US2 and US10 sites. By electron microscopy analysis, the subviral IBDV particles (SVPs) formed by VP2 were detected in the cells infected with r814UL41VP2 or r814US2VP2, but not in cells inoculated with r814US10VP2, indicating that large amount of VP2 is necessary for the SVPs formation in cells.

Animal experiments indicated that the protective efficacy of the recombinant vaccines is influenced by the insertion site notably. The antibody responses induced by r814UL41VP2 and r814US2VP2 were comparable, and were significantly higher than that induced by r814US10VP2. After challenge, r814US10VP2 did not provide any protection against mortality. The r814UL41VP2 fully protected against clinical symptoms and mortality, but conferred partial protection against bursal atrophy and lesions. In contrast, r814US2VP2 provided full protection, not only against the development of clinical signs and mortality, but also against bursal damage. The ineffective protection induced by r814US10VP2 could be due to the low expression of VP2, which was not sufficient for inducing a protective immune response against IBDV. However, why r814UL41VP2, which expressed VP2 at the highest level and replicated similarly *in vitro* with the parental virus, induced a lower level of protection than r814US2VP2, needs to be further studied. The results suggested that the antigen expression level was not the only factor that influences the protective efficacy of recombinant MDV vaccines, and other parameters, mainly the replication process of the virus *in vivo*, should be taken into consideration. With regard to developing a recombinant MDV1 vaccine against IBDV, the US2 site, which expressed foreign genes at a medium level, might be better than the high-expression UL41 site and the low-expression US10 site. Whether this is the case for generating recombinant MDV1 expressing other antigens needs to be determined, since the antigen characteristics and protective immunity are different among viruses.

Considering the uniquely advantageous characteristics of the attenuated MDV1 vector in the construction of recombinant vaccines against IBDV and its efficacious protection against high virulent MDV, many MDV1-vectored recombinant vaccines expressing the VP2 gene of IBDV have been constructed and evaluated during the last two decades^5^,^26^. However, attempts to elicit complete protection against vvIBDV infection in chickens using recombinant MDV1 vaccines have not been successful. Previously reported recombinant viruses expressing VP2 could hardly protect against bursal lesions when challenged with vvIBDV, although they protected chickens against mortality. Tsukamoto *et al*. produced a recombinant MDV1 virus expressing VP2 using the CVI988 vaccine strain as the vector; this recombinant CVI988 protected chickens against vvIBDV-induced clinical signs and mortality, but only 55% chickens were protected against bursal lesions^5^. Zhou *et al*. developed a recombinant CVI988 vaccine expressing IBDV VP2, which conferred 73% protection against vvIBDV challenge in SPF chickens and 87% protection in commercial chickens, even with a high vaccination dose of 10^4^ PFU per chicken^26^. In our study, we constructed a recombinant MDV1 expressing IBDV VP2 using a Chinese MDV1 vaccine strain, 814, as the viral vector. By cloning the VP2 gene under control of a strong promoter and inserting the VP2 cassette into the US2 gene, the r814US2VP2 vaccine fully protected chickens not only against developing clinical signs and mortality but also against the formation of bursal lesions when challenged with vvIBDV. The virulence of the HLJ0504 strain we used in the challenge experiment was previously shown to be notably higher than that of the European reference vvIBDV strains^27^. After challenged with HLJ0504, 87% chickens died in the unvaccinated challenge group; the mortality was higher than that reported in the previous studies^5^,^26^, indicating a higher pathogenic vvIBDV strain might be used here. Tsukamoto *et al*. constructed a recombinant HVT vaccine expressing VP2, which was shown to be capable of inducing complete protection against lethal IBDV challenge^22^. However, HVT vector is inferior to the attenuated MDV1 vaccine strains considering its limited protection against the prevalent high virulent MDV strains^23^,^24^,^25^. Since the attenuated MDV1 vaccine strain 814 was used here as the vector expressing VP2, the r814US2VP2 also exhibits full protection against vvMDV lethal challenge. The results indicate that a more efficacious MDV1-vectored bivalent vaccine against both vvMDV and vvIBDV infection in chickens was constructed in this study.

Over the last two decades, much attention has been paid to MDV-vectored vaccines^4^,^5^,^6^,^7^. However, recombinant MDVs have been constructed exclusively using traditional homologous recombination^24^ or the bacterial artificial chromosome (BAC) system^28^,^29^. These methods require either several rounds of plaque purification or the time-consuming excision of the BAC sequence^30^,^31^. In a methodological improvement, Reddy *et al*. developed a cosmid-based system for the rescue of vvMDV Md5 strain^32^. However, the cosmid system has not been used to construct recombinant MDV vaccines; only the generation of Md5 has been reported. The unwieldiness of the cosmid system may be due to the instability of the cosmid clones containing large DNA inserts during routine maintenance and propagation^33^. To overcome the above limitations, fosmid, which is derived from the bacterial F episome and is present in single or low copy number per cell, was introduced as a substitute for the conventional cosmid in genome library construction for its better stability and inducible copy number^33^. The fosmid technology was also used in the construction of recombinant duck enteritis virus expressing the HA gene of avian influenza virus with high efficiency^34^. Here, the complete genome of MDV 814 strain was cloned as overlapping fosmid DNAs, and the rescued virus was proven infectious with good immunogenicity. We next modified the fosmids by inserting a dual selection marker flanked by the attR1 and attR2 sequences into three different sites in MDV genome, and constructed a versatile entry vector with a multiple clone sequence flanked by the attL1 and attL2 sequences. With this system, we could generate the recombinant MDVs within two weeks after transfection without the need for repeated rounds of plaque purification and introduction of marker genes. Hence, this system is more efficient than the traditional methods. This technology is highly valuable for constructing recombinant MDVs against viruses that easily undergo antigenic drift, as vaccine update would be easier and more feasible.

With the effective protection, the r814US2VP2 vaccine represents a promising bivalent vaccine against vvIBDV and vvMDV, two lethal and immunosuppressive pathogens. Further studies are worthwhile to be conducted before its ready to use in the field farms such as the required vaccination dose, the required level of immune against IBDV and the protective efficacy in commercial chickens with maternal antibodies. Furthermore, the establishment of an efficient fosmid rescue system for generating recombinant MDVs should facilitate the analyses of MDV functional genes and vaccine-induced protective immunity against the neoplastic disease.

## Material and Methods

### Ethics Statement

All animal experiments were approved by the ethical review board of Harbin Veterinary Research Institute (HVRI) of the Chinese Academy of Agricultural Sciences (CAAS) and performed in accordance with approved animal care guidelines and protocols. The animal Ethics Committee approval number is SYXK (Heilongjiang) 2011022.

### Viruses and cells

MDV1 vaccine strain 814^18^ was used as the parent virus for producing recombinant MDVs. The vvMDV Md5^35^ and vvIBDV HLJ0504^27^ strains were used as challenge viruses. MDVs were propagated in CEFs prepared from 10-day-old specific-pathogen-free (SPF) chicken embryos.

### Construction of the MDV fosmid library

MDV strain 814 was propagated in CEFs until 80–90% CPE was obtained. The viral DNA was purified from infected cells using hypotonic lysis to release virus particles followed by micrococcal nuclease treatment to degrade cellular DNA^36^. An MDV fosmid library was constructed using the Copy Control Fosmid Library Production Kit (Epicentre). The viral DNA was sheared into 25~50 kb fragments. After blunt treatment and phosphorylation of the ends, the viral DNA was selected for 36–48 kb fragments with pulsed field gel electrophoresis (PFGE, Bio-Rad). The collected fragments were subsequently inserted into the cloning-ready fosmid vector pCC1FOS. The recombinant fosmids were packaged using MaxPlax Lambda Packaging Extracts and plated on EPI300-T1 plating cells (Epicentre). The presence of the DNA fragment inserts in these fosmids was confirmed by end sequencing with a pair of specific primers (5′-GGA TGT GCT GCA AGG CGA TTA AGT TGG-3′ and 5′-CTC GTA TGT TGT GTG GAA TTG TGA GC-3′).

### Construction of recombinant fosmids containing VP2 gene

Five fosmids (195, 214, 14, 96, and 103), which contain sequences spanning the entire genome of MDV1 strain 814, were selected for rescuing the parental virus (Fig. 2A). To facilitate the insertion of foreign genes into MDV genome, the fosmids were modified by inserting a dual selected maker open reading frame encoding the kanamycin resistance (*KanR*) gene and the toxin *ccdB* gene, flanked by the attR1 and attR2 sequences, into the MDV genome at the UL41, US10 and US2 sites. The *KanR* and ccdB-attR2 sequences were amplified from pMOD6 and pDEST22 plasmid. The aatR1-KanR-ccdB-aatR2 cassette was then combined by overlapping PCR, and 50 nucleotides matching the sequences of different areas of UL41, US10, and US2 of the 814 strain genome were introduced into the two ends of the cassette. The resultant cassettes with homology arms were inserted into 14 and 103 with Counter-Selection BAC modification kit (Gene Bridges). To simplify the insertion of foreign genes into MDV genome, a versatile entry vector pENTR-mcs was constructed by replacing the *gus* gene flanked by attL1 and attL2 in the plasmid pENTR-gus (Invitrogen) with a multiple cloning sequence.

For *VP2* expression, *VP2* from the vvIBDV strain HLJ0504 was cloned into pCAGGS plasmid to form the VP2-expressing cassette. The VP2 cassette was then inserted into pENTR-mcs. To insert the VP2 cassette into the desired sites in MDV1 genome, pENTR-VP2 was mixed with the modified fosmids and treated with LR Clonase II enzyme (Invitrogen). The mixtures were transformed into *Escherichia coli* EPI300-T1 cells.

### Rescue and characterization of the recombinant MDVs

The five fosmids with or without VP2 insertions that covered the entire MDV genome were used for virus rescue. Viral DNA inserts were released from purified fosmids by digestion with NotI and purified before transfection. Two micrograms of each fosmid DNA were used to transfect primary CEFs in 60-mm dishes by using the calcium phosphate procedure^37^. The cytopathic effects (CPE)-positive samples were harvested and characterized by electron microscopy. To verify that the VP2 gene was inserted at the desired sites, viral genomic DNAs were isolated and analysed by PCR and sequencing.

### Characterization of rescued MDVs

CEF cells infected with parental or recombinant MDVs were first characterized with electron microscopy after CPE appeared. Genomic DNA was extracted from the infected cells and digested with HindIII for pulsed-field gel electrophoresis analyses. To verify that the VP2 gene was inserted into MDV genome at the desired site, genomic DNA was isolated from the rescued viruses and analyzed by PCR with the forward primer specific to VP2 and the reverse primer matching US2. The PCR products were purified and sequenced.

### Confirmation of VP2 gene expression

The expression of VP2 from the recombinant MDVs was confirmed by IFA and western blotting with anti-VP2 MAb as the primary antibody and goat anti-mouse IgG conjugated with fluorescein isothiocyanate (FITC) or DyLight 800 as the secondary antibody. To compare the VP2 expression levels, CEF cells were infected with 1,000 PFU of the recombinant MDVs and maintained for 120 h before harvesting. After fixed and perforated, the cells were incubated first with mouse anti-VP2 MAb and then with FITC-conjugated goat anti-mouse IgG antibody. The mean fluorescence intensity (MFI) of VP2-positive cells (10,000 events were captured per sample) was assessed by FACS analysis (FACSAria; Becton Dickinson). To determine whether VP2 expressed by the recombinant MDV self-assembled into SVPs, negative-stain electron microscopy was performed as previously described^38^. Infected cell lysates were negatively stained with a 2% uranyl acetate aqueous solution, and observed using a HITACHI H-7650 electron microscope. Digital images were processed using the iTEM software (Munster, Germany).

### Stability and growth properties of the rescued viruses

To evaluate the genetic stability of the recombinant viruses, viruses were passaged in CEFs 20 times. The inserted VP2 gene was detected by using PCR and sequencing. VP2 expression was confirmed with fluorescence assays as described above. To investigate the growth properties of the recombinant MDVs, cells cultured in 6-well plates were inoculated with 100 PFU of different viruses and harvested at different time points. The serial dilutions were inoculated onto fresh CEFs seeded on 6-well plates; plaques of the different dilutions were counted five days later.

### Protection against vvIBDV challenge

To evaluate the protective efficacy of the recombinant viruses against vvIBDV, each of 15 1-day-old SPF chickens were vaccinated with 5,000 PFU of the recombinant MDVs or the parental virus, and challenged with 10^5^ EID_50_ of vvIBDV HLJ0504 at 28 days post vaccination. The clinical signs were quantified using a previously described symptomatic index^27^. The dead and surviving chickens were evaluated for bursal atrophy with the bursa: body-weight index (BBIX)^38^. Bursae with a BBIX lower than 0.70 were considered atrophied^39^. The bursae were further examined histopathologically and recorded using the histopathological bursal lesion score (HBLS)^40^. A HBLS value of no more than 1 (no or slight lesion) was defined as protected against IBDV challenge.

### Serological tests

The serum samples collected weekly were tested by ELISA (IDEXX, Westbrook, Maine). For virus neutralization (VN) assay, triplicates of serum samples were diluted serially by two-fold and mixed with equal volume of 100 TCID_50_ of cell culture-adapted HLJ0504 virus. After 60 min of incubation, the mixtures were added to CEFs followed by further incubation for 3 days. The VN titre was determined as the highest dilution at which there were no visible cytopathic effects.

### Protection against vvMDV challenge

Each of 20 1-day-old SPF chickens was vaccinated subcutaneously with 2,000 PFU of the recombinant MDVs, and challenged intraperitoneally 7 days later with 1,000 PFU of the vvMDV strain Md5. The chickens were examined for clinical signs and mortality for 12 weeks after the challenge. Both dead and surviving chickens were subjected to gross and histopathological observations for MD lesions in the liver, kidneys, spleen, nerves, and skin. The protection index (PI) was calculated as described previously^25^.

### Statistical analysis

All data were presented as the mean ± standard deviation (S.D.). One-way ANOVA was employed to evaluate the statistical differences among groups using SPSS 17.0 (SPSS Inc., Chicago, IL). Statistical significance was set at *P* < 0.05 for all tests.

## Additional Information

**How to cite this article:** Li, K. *et al*. Recombinant Marek’s disease virus type 1 provides full protection against very virulent Marek’s and infectious bursal disease viruses in chickens. *Sci. Rep.* **6**, 39263; doi: 10.1038/srep39263 (2016).

**Publisher's note:** Springer Nature remains neutral with regard to jurisdictional claims in published maps and institutional affiliations.

## Supplementary Material

Supplementary Information

## References

[b1] Marek J. Multiple Nervenentzündung (Polyneuritis) bei Hühnern. Dtsch. Tierärztl. Wochenschr. 15, 417–421 (1907).

[b2] Biggs P. M. & Nair V. The long view: 40 years of Marek’s disease research and avian pathology. Avian Pathol. 41, 3–9 (2012). 22845316 10.1080/03079457.2011.646238

[b3] Venugopal K. Marek’s disease: an update on oncogenic mechanisms and control. Res. Vet. Sci. 69, 17–23 (2000). 10924389 10.1053/rvsc.2000.0396

[b4] Sakaguchi M. . Protection of chickens with or without maternal antibodies against both Marek’s and Newcastle diseases by one-time vaccination with recombinant vaccine of Marek’s disease virus type 1. Vaccine 16, 472–479 (1998). 9491501 10.1016/s0264-410x(97)80001-1

[b5] Tsukamoto K. . Protection of chickens against very virulent infectious bursal disease virus (IBDV) and Marek’s disease virus (MDV) with a recombinant MDV expressing IBDV VP2. Virology 257, 352–362 (1999). 10329546 10.1006/viro.1999.9641

[b6] Zhang X., Wu Y., Huang Y. & Liu X. Protection conferred by a recombinant Marek’s disease virus that expresses the spike protein from infectious bronchitis virus in specific pathogen-free chicken. Virol. J. 9, 85 (2012). 22559869 10.1186/1743-422X-9-85 PMC3447679

[b7] Zhang F. . Transcriptional activity comparison of different sites in recombinant Marek’s disease virus for the expression of the H9N2 avian influenza virus hemagglutinin gene. J. Virol. Methods 207, 138–145 (2014). 25034124 10.1016/j.jviromet.2014.07.011

[b8] van den Berg T. P. Acute infectious bursal disease in poultry: a review. Avian Pathol. 29, 175–194 (2000). 19184804 10.1080/03079450050045431

[b9] Jackwood D. H. & Saif Y. M. Antigenic diversity of infectious bursal disease viruses. Avian Dis. 31, 766–770 (1987). 2831868

[b10] Chettle N., Stuart J. C. & Wyeth P. J. Outbreak of virulent infectious bursal disease in East Anglia. Vet. Rec. 125, 271–272 (1989). 2552640 10.1136/vr.125.10.271

[b11] Hsieh M. K., Wu C. C. & Lin T. L. DNA-mediated vaccination conferring protection against infectious bursal disease in broiler chickens in the presence of maternal antibody. Vaccine 28, 3936–3943 (2010). 20394722 10.1016/j.vaccine.2010.03.066

[b12] Giambrone J. J. . Effect of infectious bursal agent on the response of chickens to Newcastle disease and Marek’s disease vaccination. Avian Dis. 20, 534–544 (1976). 183651

[b13] Muskett J. C., Reed N. E. & Thornton D. H. Increased virulence of an infectious bursal disease live virus vaccine after passage in chicks. Vaccine 3, 309–312 (1985). 2998114 10.1016/s0264-410x(85)90161-6

[b14] Coulibaly F. . The birnavirus crystal structure reveals structural relationships among icosahedral viruses. Cell 120, 761–772 (2005). 15797378 10.1016/j.cell.2005.01.009

[b15] Fahey K. J., Erny K. & Crooks J. A conformational immunogen on VP2 of infectious bursal disease virus that induces virus-neutralizing antibodies that passively protect chickens. J. Gen. Virol. 70, 1473–1481 (1989). 2543787 10.1099/0022-1317-70-6-1473

[b16] Haq K., Schat K. A. & Sharif S. Immunity to Marek’s disease: where are we now? Dev. Comp. Immunol. 41, 439–446 (2013). 23588041 10.1016/j.dci.2013.04.001

[b17] Cui H. Y. . Construction of an infectious Marek’s disease virus bacterial artificial chromosome and characterization of protection induced in chickens. J. Virol. Methods 156, 66–72 (2009). 19026690 10.1016/j.jviromet.2008.10.021

[b18] Zhang F. . Comparative full-length sequence analysis of Marek’s disease virus vaccine strain 814. Arch. Virol. 157, 177–183 (2012). 21984218 10.1007/s00705-011-1131-8

[b19] Cui H. . Avirulent Marek’s disease virus type 1 strain 814 vectored vaccine expressing avian influenza (AI) virus H5 haemagglutinin induced better protection than turkey herpesvirus vectored AI vaccine. PLoS One 8, e53340 (2013). 23301062 10.1371/journal.pone.0053340 PMC3536743

[b20] Li Y. . Recombinant herpesvirus of turkeys as a vector-based vaccine against highly pathogenic H7N1 avian influenza and Marek’s disease. Vaccine 29, 8257–8266 (2011). 21907750 10.1016/j.vaccine.2011.08.115

[b21] Reddy S. K. . Protective efficacy of a recombinant herpesvirus of turkeys as an in ovo vaccine against Newcastle and Marek’s diseases in specific-pathogen-free chickens. Vaccine 14, 469–477 (1996). 8782342 10.1016/0264-410x(95)00242-s

[b22] Tsukamoto K. . Complete, long-lasting protection against lethal infectious bursal disease virus challenge by a single vaccination with an avian herpesvirus vector expressing VP2 antigens. J. Virol. 76, 5637–5645 (2002). 11991992 10.1128/JVI.76.11.5637-5645.2002 PMC137028

[b23] Bülow V. & Biggs P. M. Differentiation between strains of Marek’s disease virus and turkey herpesvirus by immunofluorescenece assays. Avian Pathol. 4, 133–146 (1975). 18777301 10.1080/03079457509353859

[b24] Darteil R. . Herpesvirus of turkey recombinant viruses expressing infectious bursal disease virus (IBDV) VP2 immunogen induces protection against an IBDV virulent challenge in chickens. Virology 211, 481–490 (1995). 7645252 10.1006/viro.1995.1430

[b25] Sonoda K. . Development of an effective polyvalent vaccine against both Marek’s and Newcastle diseases based on recombinant Marek’s disease virus type 1 in commercial chickens with maternal antibodies. J. Virol. 74, 3217–3226 (2000). 10708438 10.1128/jvi.74.7.3217-3226.2000 PMC111822

[b26] Zhou X. . Protection of chickens, with or without maternal antibodies, against IBDV infection by a recombinant IBDV-VP2 protein. Vaccine 28, 3990–3996 (2010). 20338216 10.1016/j.vaccine.2010.03.021

[b27] Li K. . Genetic, antigenic and pathogenic characterization of four infectious bursal disease virus isolates from China suggests continued evolution of very virulent viruses. Infect. Genet. Evol. 30, 120–127 (2015). 25528137 10.1016/j.meegid.2014.12.016

[b28] Petherbridge L. . Replication-competent bacterial artificial chromosomes of Marek’s disease virus: novel tools for generation of molecularly defined herpesvirus vaccines. J. Virol. 77, 8712–8718 (2003). 12885890 10.1128/JVI.77.16.8712-8718.2003 PMC167215

[b29] Baigent S. J. . Herpesvirus of turkey reconstituted from bacterial artificial chromosome clones induces protection against Marek’s disease. J. Gen. Virol. 87, 769–776 (2006). 16528024 10.1099/vir.0.81498-0

[b30] Wagner M., Jonjic S., Koszinowski U. H. & Messerle M. Systematic excision of vector sequences from the BAC-cloned herpesvirus genome during virus reconstitution. J. Virol. 73, 7056–7060 (1999). 10400809 10.1128/jvi.73.8.7056-7060.1999 PMC112796

[b31] Zhao Y., Petherbridge L., Smith L. P., Baigent S. & Nair V. Self-excision of the BAC sequences from the recombinant Marek’s disease virus genome increases replication and pathogenicity. Virol. J. 5, 19 (2008). 18230192 10.1186/1743-422X-5-19 PMC2248170

[b32] Reddy S. M. . Rescue of a pathogenic Marek’s disease virus with overlapping cosmid DNAs: use of a pp38 mutant to validate the technology for the study of gene function. Proc. Natl. Acad. Sci. USA 99, 7054–7059 (2002). 11997455 10.1073/pnas.092152699 PMC124527

[b33] Kim U. J., Shizuya H., de Jong P. J., Birren B. & Simon M. I. Stable propagation of cosmid sized human DNA inserts in an F factor based vector. Nucleic Acids Res. 20, 1083–1085 (1992). 1549470 10.1093/nar/20.5.1083 PMC312094

[b34] Liu J. . A duck enteritis virus-vectored bivalent live vaccine provides fast and complete protection against H5N1 avian influenza virus infection in ducks. J. Virol. 85, 10989–10998 (2011). 21865383 10.1128/JVI.05420-11 PMC3194986

[b35] Tulman E. R. . The genome of a very virulent Marek’s disease virus. J. Virol. 74, 7980–7988 (2000). 10933706 10.1128/jvi.74.17.7980-7988.2000 PMC112329

[b36] Volkening J. D. & Spatz S. J. Purification of DNA from the cell-associated herpesvirus Marek’s disease virus for 454 pyrosequencing using micrococcal nuclease digestion and polyethylene glycol precipitation. J. Virol. Methods 157, 55–61 (2009). 19103224 10.1016/j.jviromet.2008.11.017

[b37] Morgan R. W., Cantello J. L. & McDermott C. H. Transfection of chicken embryo fibroblasts with Marek’s disease virus DNA. Avian Dis. 34, 345–351 (1990). 2164390

[b38] Dubochet J. . Cryo-electron microscopy of vitrified specimens. Q. Rev. Biophys. 21, 129–228 (1988). 3043536 10.1017/s0033583500004297

[b39] Li K. . Codon optimization and woodchuck hepatitis virus posttranscriptional regulatory element enhance the immune responses of DNA vaccines against infectious bursal disease virus in chickens. Virus Res. 175, 120–127 (2013). 23631937 10.1016/j.virusres.2013.04.010

[b40] Lucio B. & Hitcher, B. Immunosuppression and active response induced by infectious bursal disease virus in chickens with passive antibodies. Avian Dis. 24, 189–196 (1979).

