# Supplementary material for: Recombinant Marek’s disease virus type 1 provides full protection against very virulent Marek’s and infectious bursal disease viruses in chickens
Source: Sci Rep. 2016 Dec 16;6:39263. doi: 10.1038/srep39263 (PMC5159867; doi:10.1038/srep39263)
Supplement: Supplementary Information [file srep39263-s1.pdf]

Recombinant Marek's disease virus type 1 provides full protection against very virulent Marek's and infectious bursal disease viruses in chickens

Kai Li, Yongzhen Liu, Changjun Liu, Li Gao, Yanping Zhang, Hongyu Cui, Yulong Gao, Xiaole Qi, Li Zhong, Xiaomei Wang*

Avian Immunosuppressive Diseases Division, State Key Laboratory of Veterinary Biotechnology, Harbin Veterinary Research Institute, Chinese Academy of Agricultural Sciences, Harbin 150069, P.R. China

E-mail address: likai01@caas.cn (K.L.); yongzhenhvri@163.com (Y.L.); liucj93711@hvri.ac.cn (C.L.); gaoli0820@163.com (L.G.); zhyp_77@hvri.ac.cn (Y.Z.); cuihongyu@caas.cn (H.C.); ylg@hvri.ac.cn (Y.G.); qxl@hvri.ac.cn (X.Q.); lizimd@163.com (L.Z.); xmw@hvri.ac.cn (X.W.).

***Corresponding author**. Tel: +86-451-51051694; Fax: +86-451-51997166.

E-mail address: xmw@hvri.ac.cn.

Address: 678 Haping Road, Harbin, Heilongjiang 150069, P.R. China.

Table S1 Generation of MDV1 vaccine strain 814 from overlapping fosmid DNAs.

(A) Sizes of fosmid viral DNA fragments and their corresponding locations within the MDV1 vaccine strain 814 genome.

Fosmid	Location in 814 genome (5'-3')	Size	Fosmid	Location in 814 genome (5'-3')	Size
**195**	1-47873	47873	126	95908-139279	43372
131	1-39808	39808	36	99707-139612	39906
277	35972-74931	38960	139	100217-139425	39210
279	36841-76702	39862	85	100139-139157	39019
179	36994-78457	41464	**96**	106337-139612	33277
**214**	40028-79118	39091	115	115335-150262	34928
116	41974-81299	39326	4	114047-146395	32349
163	66861-104662	37802	**103**	129115-172541	43427
38	68595-104925	36331	219	132297-172541	40245
133	70330-108104	37775	112	134376-731	39987
145	71907-108935	37029	2	138630-6922	40854
**14**	72447-113806	41360	203	138759-7486	41269

(B) Fosmid combinations used for virus generation. More "+" represent more plaques were produced in CEFs.

Set	Fosmid combinations	CPE
1	195+116+14+96+219	++++
**2**	**195+214+14+96+103**	**+++++**
3	195+179+145+85+112	++++
4	195+279+133+139+219	+++
5	195+277+38+36+103	++++
6	195+116+145+139+112	+++
7	195+214+133+85+103	++++
8	131+179+133+139+2	++
9	131+277+163+126+115+2	++
10	131+279+38+36+4+203	++

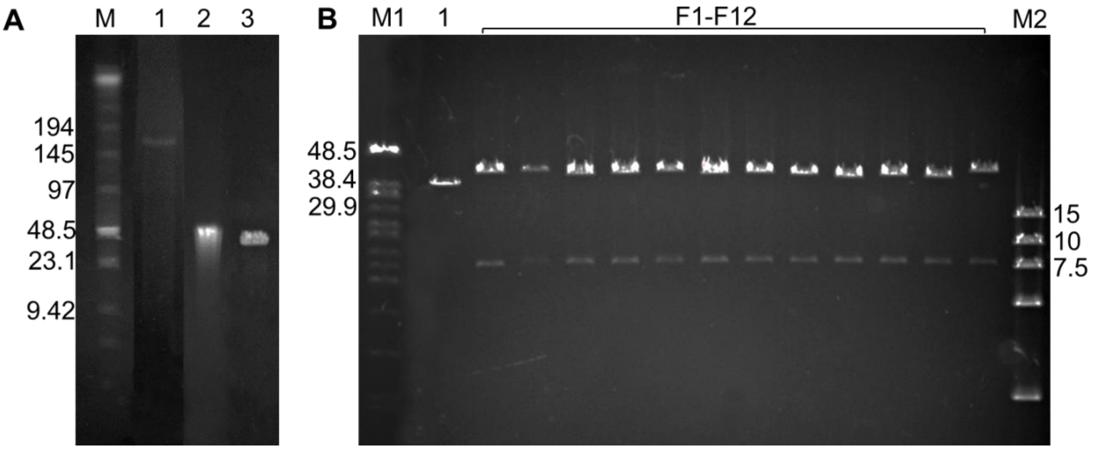

Figure S1. Construction of the genome fosmid library of the MDV1 vaccine strain 814. (A) Purification and size selection of the MDV viral DNAs. M, Bio-Rad PFGE marker; 1, purified MDV genomic DNA; 2, sheared and end-repaired DNA fragments; 3, recovered fragments from PFGE gels with size of 36-48 kb. **(B)** Size determination of the fragments inserted in the fosmid vector by NotI digestion. M1, CHEF DNA size standard; M2, DL15000 marker; 1, 36 kb control DNA; F1-F12, recombinant fosmid clones digested with NotI.

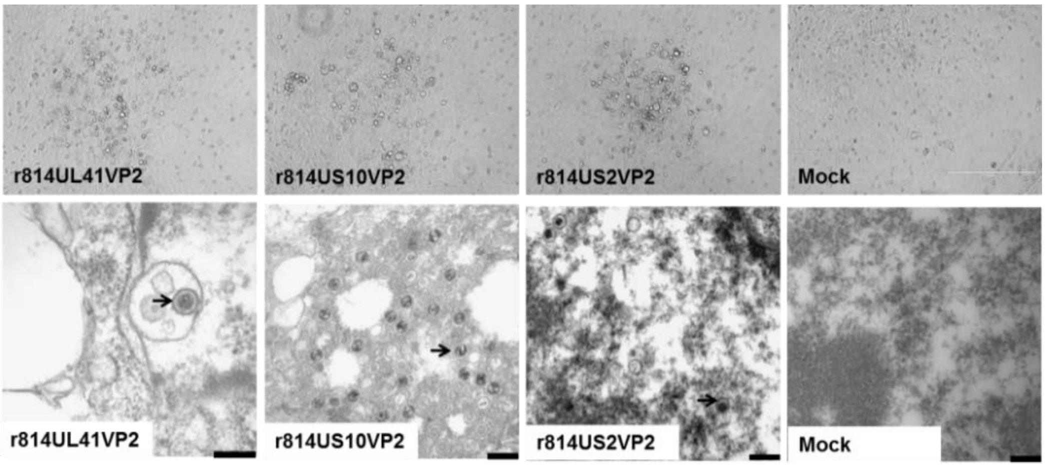

Figure S2. Rescue of the recombinant MDVs containing VP2 gene. The cytopathic effects and electron microscopy detection of the rescued viruses in CEFs. Arrows represent the MDV virions detected in the cell nucleus in infected cells. Bar length, 200 nm.
